# Comparison of the Effects of Elastic and Rigid Taping on Gross Motor Function, Balance, and Functional Capacity in Children with Hemiplegic Cerebral Palsy: A Randomized, Single-Blinded Trial

**DOI:** 10.3390/children12111551

**Published:** 2025-11-17

**Authors:** Duygu Korkem Yorulmaz, Rıdvan Gök, Emine Handan Tüzün, Duygu Türker, Buse Birbir, Tezel Yıldırım Şahan

**Affiliations:** 1 Gulhane Faculty of Physiotherapy and Rehabilitation, University of Health Sciences, 06010 Ankara, Turkey; duygu.turker@sbu.edu.tr (D.T.); buse.korkmaz@sbu.edu.tr (B.B.); tezelyildirim.sahan@sbu.edu.tr (T.Y.Ş.); 2 Institute of Health Sciences, Uskudar University, 34662 Istanbul, Turkey; fztridvan27@gmail.com; 3 Department of Physiotherapy and Rehabilitation, Faculty of Health Sciences, Eastern Mediterranean University, Famagusta 99628, Cyprus; handan.tuzun@emu.edu.tr

**Keywords:** cerebral palsy, motor function, postural balance, functional capacity, taping, physiotherapy

## Abstract

**Highlights:**

**What are the main findings?**

- This is the first trial comparing Kinesio taping and rigid taping in children with hemiplegic cerebral palsy.
- Both taping types improved motor function, walking, balance, and functional capacity.

**What is the implication of the main finding?**

- Taping can be used as an effective adjunct in pediatric neurorehabilitation to enhance functional outcomes.

**Abstract:**

**Background/Objectives:** This randomized, single-blinded trial compared the effects of Kinesio taping (KT) and rigid taping (RT) on gross motor function, balance, and functional capacity in children with hemiplegic cerebral palsy (HCP). **Methods:** Fifty-two children (aged 7–16) were assessed using the Gross Motor Function Measure (GMFM), Pediatric Berg Balance Scale (PBBS), Time-Up-and-Go (TUG), and 2-Minute Walk Test (2-MWT). **Results:** Both KT and RT produced significant intra-group improvements in GMFM, PBBS, TUG, and 2-MWT scores (*p* ≤ 0.001). Although nonparametric analysis suggested greater changes for KT in TUG and 2-MWT (*p* < 0.001; *p* = 0.036), no significant inter-group differences were found when baseline scores were adjusted using the General Linear Model (GLM) (2-MWT: *p* = 0.29; TUG: *p* = 0.087). **Conclusions:** KT and RT are similarly effective adjuncts to physiotherapy, improving gross motor function, balance, and functional capacity in children with HCP. Therefore, the choice between KT and RT may be guided by clinical preference, child tolerance, and therapeutic goals rather than superiority of effect.

## 1. Introduction

Hemiplegic Cerebral Palsy (HCP) is a subtype characterized by motor impairments affecting the arm and leg on one side of the body. Among children born at term, HCP is the most common presentation of cerebral palsy; however, among preterm children, it is second only to diplegic cerebral palsy [1]. Children with HCP commonly experience increased muscle tone, muscle weakness, sensory deficits, and reduced selective motor control. These primary impairments contribute to secondary complications, including deficits in postural control and balance, adversely impacting self-care, mobility, social participation, and overall quality of life [2,3].

The foot and ankle play a pivotal role in maintaining balance by providing somatosensory feedback in the standing position and serving as key sensory organs during walking [4]. In children with HCP, motor impairments on the affected side often result in spasticity of the ankle plantar flexors and weakness of the antagonist dorsiflexors. This imbalance disrupts joint biomechanics, leading to ankle instability and impairments in posture, balance, and gait control [5,6]. Damiano et al. emphasize that achieving proper lower-limb alignment and controlling foot position during the stance phase are critical treatment goals for children with HCP [7].

To address these challenges, physiotherapy interventions, including taping, improve postural alignment, joint stability, muscle activation, spasticity management, and sensory stimulation. Taping techniques are widely used in clinical practice and broadly classified as rigid taping (RT) and elastic taping (Kinesio Taping, KT). KT stimulates muscle activation, mechanoreceptors, and fascia while allowing joint movement. It has been proposed that KT applications in children with cerebral palsy provide proprioceptive and tactile input, optimize muscle length, stabilize hypermobile joints, and enhance both static and dynamic balance. KT may also improve sitting balance and functional performance without restricting joint movement, though the current evidence supporting these claims remains limited [8,9]. Meta-analyses of KT in children with CP report moderate effect sizes for gross motor function, balance, and muscle tension. Still, heterogeneity and variable protocols limit interpretation. Despite promising outcomes, the evidence base remains inconclusive [10]. Systematic reviews note mixed results. Da Costa et al. observed improved dynamic activities but no benefit in static balance with KT in four children with HCP [11]. Guchan & Mutlu and Cunha et al. concluded that evidence across pediatric CP populations is weak and inconsistent, and Pavão et al. found no change in postural balance following KT. Reviewers further emphasize considerable heterogeneity in taping methods (limb vs. trunk, tension, duration), age groups, and outcome measures [12,13,14]. RT, often referred to as athletic tape, is used to correct malalignment and support extremities by reducing pain, providing proprioceptive feedback during activity, and limiting excessive joint motion. While RT offers robust sensory input through its firm application, its lack of elasticity may irritate the skin and limit its duration of use. Additionally, research indicates that RT works better than KT at regulating joint movement [15]. Although therapeutic taping has been widely studied in children with CP, the majority of research has focused on KT. In contrast, RT has been used primarily in sports medicine and remains relatively under-investigated in pediatric neurorehabilitation, particularly in children with CP. The limited number of randomized controlled trials specifically testing RT protocols in this population, along with substantial heterogeneity in tape types, application methods, and outcome measures, highlights a clear gap in the literature that the present study aims to address [16].

Thus, there is a clear gap in the current literature. First, there is a lack of head-to-head randomized controlled trials directly comparing the effects of KT and RT in HCP. Second, the existing evidence regarding the effectiveness of KT specifically in children with HCP is limited and inconsistent, making it difficult to draw definitive clinical conclusions. Third, previous studies vary substantially in terms of taping protocols, such as the degree of tension applied, anatomical site of application, duration of use, and outcome measures, highlighting the absence of standardized intervention guidelines in this field [8,9,10,12].

Based on this gap in the literature, the present study aims to compare the effects of KT and RT on gross motor functions, balance, and functional capacity in children with HCP.

By directly comparing two clinically relevant taping modalities under standardized conditions, this study seeks to clarify their relative efficacy and inform evidence-based physiotherapy practice in pediatric neurorehabilitation.

## 2. Materials and Methods

### 2.1. Trial Design

The study was designed as a two-arm, single-blinded, randomized trial comparing the effects of KT and RT in children with hemiplegic cerebral palsy and was conducted and reported in accordance with the CONSORT guidelines for randomized trials.

### 2.2. Participants

The inclusion criteria for the study were: (i) presence of a diagnosis of HCP, (ii) being between the ages of 7 and 18, and (iii) having GMFCS levels I or II. The study focused on children with GMFCS levels I–II, as these levels represent ambulatory individuals with sufficient motor control to perform the functional tasks and gait assessments required by the protocol. Including children with higher GMFCS levels could have introduced variability related to severe motor limitations rather than the effects of taping itself. Exclusion criteria were: (i) having additional neurological diagnoses affecting balance or gross motor function, (ii) having severe cognitive impairment, and (iii) having recent orthopedic surgery or botulinum toxin injection (within the past six months). The study involved children with hemiparetic cerebral palsy (HCP) aged 7–16 years who presented to the Special Child Therapy, Special Education and Rehabilitation Center and voluntarily participated between January 2023 and January 2024.

**Figure 1:**
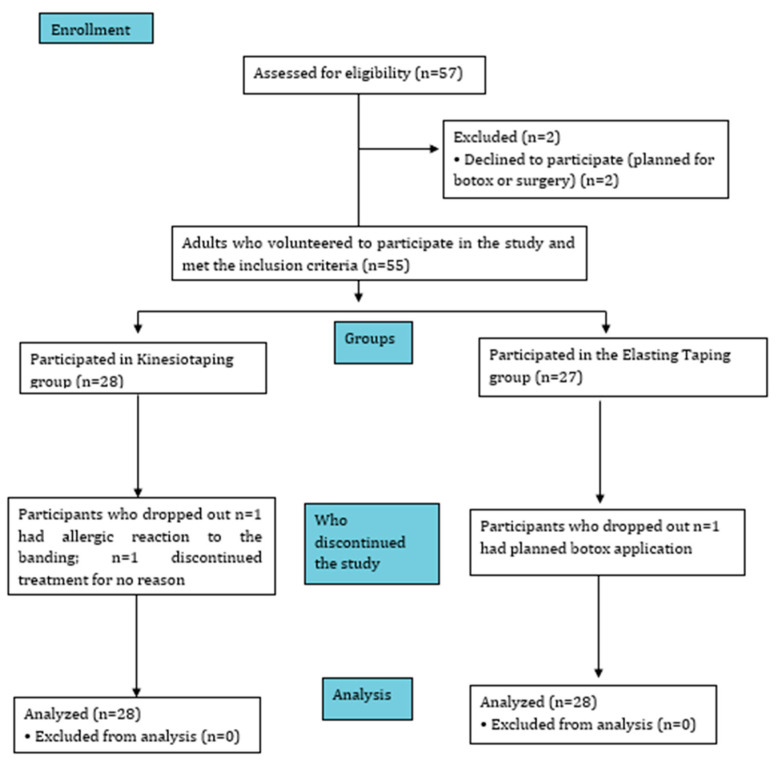
Flow Diagram of the Study.

The sample size was calculated using G*Power 3.1.9.2 software, considering statistical tests and Cohen’s recommended effect size values for behavioral sciences [17]. According to the study conducted by Tabataee et al., strength analysis measurement was made based on the results of the timed get-up-and-walk test as the primary outcome measurement. In the 12-week treatment period, this number was reached, considering a 20% loss of cases [18]. Assuming a two-tailed Mann–Whitney U test for group comparisons, with α = 0.05, β = 0.20, and d = 0.8, a total sample size of 52 participants (26 per group) was determined (Figure 1).

Participants were randomly assigned to one of two intervention groups (KT or RT) in a 1:1 ratio using a computer-generated randomization sequence created with Random Allocation Software (version 1.0). Block randomization with a fixed block size of 4 was employed to ensure equal distribution of participants between the groups.

The randomization sequence was generated by an independent researcher who was not involved in participant recruitment, assessment, or intervention delivery. To maintain allocation concealment, the group assignments were placed in sequentially numbered, opaque, sealed envelopes. Each envelope was opened only after a participant provided written informed consent and completed the baseline assessment.

As a result, 26 participants were allocated to the KT and 26 participants to the rigid taping group (RT).

**Figure 2:**
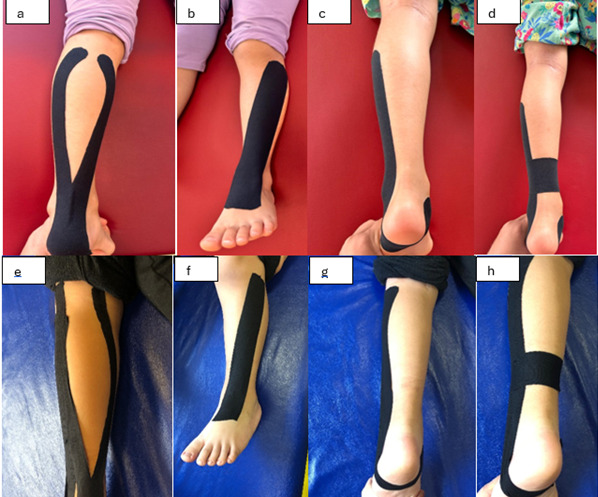
Standardized taping applications in the KT and RT groups. (**a**) Gastrocnemius inhibition, (**b**) dorsiflexion facilitation, (**c**) ground contact correction, (**d**) peroneal muscle activation with KT; (**e**–**h**) corresponding applications with RT in a neutral ankle position.

All taping procedures were performed by the same physiotherapist (Figure 2).

All outcome assessments were conducted at baseline and after the 12-week intervention period by a physiotherapist who was blinded to group allocation and was not involved in the treatment procedures or randomization process. To maintain blinding, participants were instructed not to disclose any information about the type of taping they received during the assessment sessions. Additionally, the assessor was not present during the intervention sessions and had no access to group allocation records or intervention schedules.

The success of the blinding procedure was monitored by ensuring that the assessor remained unaware of group identities throughout the study period. No instances of unblinding were reported.

Ethical approval was obtained from Istinye University Non-Clinical Research Ethics Committee (430, 02, 23 February 2018). Written informed consent was obtained from the families of all participating children before the study.

### 2.3. Outcome Measures

Sociodemographic data, including age and gender, were recorded. The Gross Motor Function Classification System (GMFCS) was used to classify the gross motor function levels of the participants. Motor function was assessed using the Gross Motor Function Measure-66 (GMFM-66), a validated and reliable tool comprising 66 items across five dimensions: lying and rolling (A = 4), sitting (B = 15), crawling and kneeling (C = 10), standing (D = 13), and walking, running, and jumping (E = 24). The GMFM-66 has been widely used to evaluate changes in motor performance in children with cerebral palsy, including those with hemiplegic subtypes [19,20].

Functional balance was evaluated using the Pediatric Berg Balance Scale (PBBS), a validated adaptation of the Berg Balance Scale for children with cerebral palsy. The PBBS consists of 14 items, each scored from 0 to 4, with a maximum score of 56 [21]. The PBBS has demonstrated good reliability and sensitivity in detecting balance changes in children with cerebral palsy [22].

Walking speed, postural control, balance, and functional mobility were assessed using the Timed Up and Go Test (TUG) [14]. Participants were instructed to rise from a child-sized chair upon the command “Go,” walk 3 m at a comfortable speed, touch a picture placed at shoulder height, return to the chair, and sit down. Timing began when the child’s back left the chair and stopped when they returned to the seated position. The test was performed three times, with a 30 s rest period between trials to prevent fatigue, and the mean value of the three trials was used for analysis. The TUG test has been validated for children with cerebral palsy and is sensitive to changes in functional mobility [23,24].

Functional capacity was evaluated using the Two-Minute Walk Test (2-MWT), a reliable measure for assessing submaximal walking performance in pediatric populations. Children were instructed to walk at a comfortable pace for two minutes along a 15 m marked walkway, and the total distance covered was recorded. The test was performed under the supervision of the same physiotherapist to ensure safety and consistency [25]. Previous studies have reported good test–retest reliability of the 2-MWT in children with cerebral palsy [26].

To ensure standardization across assessments, all evaluations were performed by the same experienced pediatric physiotherapist in a quiet clinical setting, using the same equipment and instructions for all participants. Each test was conducted at the same time of day to minimize fatigue effects, and children wore their usual footwear and orthoses, if prescribed.

### 2.4. Interventions

To ensure consistency across participants, all taping applications were performed by the same certified physiotherapist, who had completed advanced Kinesio Taping certification and had more than five years of pediatric clinical experience. The therapist practiced the taping protocol before data collection to calibrate manual application tension and confirm reproducibility [27].

Kinesio taping techniques

1. Inhibition technique (Gastrocnemius muscle): A Y-shaped tape was applied with 15–25% tension from the posterior calcaneus to the medial and lateral gastrocnemius (Figure 2a).
2. Functional correction (Dorsiflexion facilitation): While the foot was in dorsiflexion, one end of the tape was placed in the middle of the dorsum of the foot, and the other end was placed in the middle part of the tibia without stretching. Then the foot was brought to maximum plantar flexion, and the tape was adhered (Figure 2b)
3. Correction for ground contact: The subtalar joint was positioned in an eversion position, and taping was performed starting under the medial malleolus, without tension until the lateral outer edge of the calcaneus, then with 100% tension until 10–15 cm below the fibular head. No tension was applied in the last 5 cm (Figure 2c).
4. Peroneal muscle activation: Tape was applied to the distal fibula using a correction technique (Figure 2d).

In the RT group, the non-elastic athletic tape was applied following the same anatomical guidance but without stretch, maintaining a neutral ankle position (Figure 2e–h). Both taping groups were taped for 12 weeks, 3 times per week. Children with HCP continued their daily lives with taping during the treatment period. KT is 100% cotton, acrylic adhesive, breathable, and latex-free, and RT is a zinc oxide-based hard cotton tape fabric.

All participants received a standardized physiotherapy and rehabilitation program in addition to the taping interventions. The program, administered by an experienced pediatric physiotherapist, aimed to improve motor control, balance, postural symmetry, and functional mobility. Each session lasted 45–60 min and was conducted three times per week for 12 weeks. The sessions included warm-up and stretching exercises for lower-limb muscles, neurodevelopmental activities based on the Bobath concept to facilitate selective movement control, and balance training on stable and unstable surfaces. Gait exercises focused on improving step symmetry and heel strike, while strengthening and functional activities such as sit-to-stand and stair climbing were used to enhance daily motor performance [28,29,30].

### 2.5. Statistical Analysis

Data analysis was performed using IBM SPSS Statistics V.20.0.0. The Shapiro–Wilk test revealed non-normal data distribution (*p* < 0.05), leading to nonparametric tests. Group comparisons were analyzed with the Mann–Whitney U test, while the Chi-square test assessed categorical variables. Intra-group changes were analyzed using the Wilcoxon test. In this study, General Linear Model (GLM/ANCOVA) was used to control for the effect of baseline measurements when comparing post-treatment differences between two groups.

Descriptive statistics were expressed as mean ± standard deviation or frequency and percentages. Statistical significance was set at *p* < 0.05. Differences between groups were interpreted using *p*-values and 95% confidence intervals (95% CI):

1. Group means were considered different if *p* < 0.05 and 95% CI limits did not overlap.
2. The group means were considered significantly different if the 95% CI of mean differences excluded zero [31].

Effect sizes were calculated to evaluate clinical significance [32].

## 3. Results

The mean age of the children was 10.2 ± 2.6 years. In the KT group, there were 16 males (61.5%) and 10 females (38.5%), while the RT group included 15 males (57.7%) and 11 females (42.3%). No significant differences were found between the groups regarding age (*p* = 0.817) or gender distribution (*p* = 0.777) (Table 1).

GMFCS levels were comparable between the groups (*p* = 0.405). In the KT group, 15 children (57.7%) were classified as level I, and 11 (42.3%) as level II. In the RT group, 12 children (46.2%) were at level I, and 14 (53.8%) were at level II (Table 2).

Post-treatment significant improvements were noted in both groups compared to baseline for both the D and E sections of GMFM (all *p* ≤ 0.001) (Table 2). However, no significant differences were observed between the groups in the GMFM-66 D and E section scores before and after treatment (all *p* > 0.05) (Table 3). After adjusting for baseline scores of GMFM D and E in the General Linear Model analysis, no significant difference was found between groups post-treatment (*p* = 0.551, *p* = 0.102).

The PBBS scores and results of the 2 min walk test were statistically similar between the groups, both before treatment (*p* > 0.05). After adjusting for baseline scores in the General Linear Model analysis, no significant difference was found between groups post-treatment (*p* = 0.105). However, the KT group demonstrated a significant advantage post-treatment in the 2 min walk test (*p* = 0.036) (Table 3). A statistically significant difference was observed between the groups after treatment for the TUG test (*p* < 0.05) (Table 3). After adjusting for baseline scores in the General Linear Model analysis, no significant difference was found between groups post-treatment (*p* = 0.087). The effect sizes of all evaluation parameters within the group are given in Table 2. For 2 min after adjusting for baseline scores in the General Linear Model analysis, no significant difference was found between groups post-treatment (*p* = 0.29).

In intra-group comparisons of balance and gait tests, significant improvements were observed post-treatment in the KT group for the PBBS, TUG test, and 2 min walk test scores (all *p* ≤ 0.001). Similarly, the RT group showed significant improvements post-treatment in the PBBS, TUG test, and 2 min walk test scores (all *p* ≤ 0.001) (Table 2). The effect sizes of inter-group comparisons for all evaluation parameters are presented in Table 3.

## 4. Discussion

The present study is the first randomized, single-blind trial comparing the effects of kinesiology taping (KT) and rigid taping (RT) in children with hemiplegic cerebral palsy (HCP). The findings indicate that both KT and RT improve gross motor function, static and dynamic balance, and functional capacity after a 12-week intervention compared to baseline. These results are consistent with previous research and underscore the potential benefits of taping interventions for children with HCP. The effect sizes within the group vary from moderate to very large. In particular, the effect sizes for GMFM-E, BBS, and 2 min walk in the KT group indicate a “large effect size,” which shows that KT has brought about significant improvements. However, although the unadjusted change score of the 2MWT initially appeared to favor KT, the General Linear Model (GLM) analysis showed that this difference did not remain significant after adjusting for baseline values. Therefore, the higher change score observed in the KT group may be partially attributable to variability in baseline performance rather than a true differential treatment effect.

### 4.1. Gross Motor Function

Gross motor function is a critical parameter in children with HCP [33,34]. Kemer et al. reported that a four-week KT intervention applied twice weekly positively affected gross motor function in children with HCP compared to a control group [33]. Similarly, Kaya Kara et al. examined the long-term effects of a 12-week KT intervention and observed improvements in gross motor functions. However, the improvements were not statistically significant compared to the control group. In alignment with these findings, this study demonstrated that both KT and RT interventions significantly improved gross motor function. Consistent with GLM results, no between-group differences were found, suggesting that both taping techniques produce comparable effects on gross motor outcomes. The ceiling effect of the GMFM may partially explain the lack of inter-group differences in the current study, as the majority of participants were classified as Level I or II, which limits the measure’s sensitivity to detect subtle improvements. Additionally, the similar outcomes between KT and RT suggest that both methods may offer comparable proprioceptive and neuromuscular facilitation, despite their mechanical differences.

### 4.2. Static and Dynamic Balance

Impaired balance significantly restricts children with HCP in mobility and interaction with their environment, including social engagement with peers and relatives. Tabatabaee et al. found no improvements in balance following a two-day taping intervention; however, they reported positive effects after a two-week intervention [35]. Similarly, Partoazar et al. highlighted the efficacy of KT in enhancing dynamic balance in children with HCP. This study extends these findings by demonstrating significant improvements in both static and dynamic balance after a 12-week intervention of KT and RT. These outcomes are likely due to the tactile input provided by the taping, which stimulates mechanoreceptors in the skin, enhances proprioceptive feedback, and increases balance awareness through augmented afferent input to the central nervous system [36]. Importantly, GLM analysis revealed that these improvements were comparable between KT and RT, indicating that both taping approaches can reinforce proprioceptive feedback sufficiently to promote balance gains.

### 4.3. Functional Capacity

Functional capacity in children with HCP relies on muscle strength, motor coordination, and stability during movement. Tabatabaee et al. reported positive changes in the functional capacities of children with cerebral palsy following a two-week KT intervention [18]. Similarly, Lin et al. also noted in their meta-analysis and systematic review studies that KT has a positive effect on functional capacity [10]. In children with cerebral palsy, changes in lower extremity biomechanics negatively affect walking efficiency, reducing functional capacity. Strategic application of KT can help realign biomechanical alignment. Evidence from studies evaluating walking suggests that a combination of targeted tactile stimulation of weakened muscles and inhibitory input to overactive muscles can reshape neuromuscular activation patterns, thereby improving lower extremity alignment and walking symmetry. Ghafar and colleagues emphasized in their study on cerebral palsy that the combined use of RT and KT improves walking parameters and increases functional capacity [16]. These findings demonstrated significant increases in functional capacity (TUG and 2MWT) in both groups; however, the between-group difference in 2MWT did not persist after baseline adjustment in the GLM analysis. This suggests that the apparent superiority of KT in functional capacity may have been influenced by baseline differences rather than a true differential treatment effect.

Collectively, these results indicate that KT and RT exert similar therapeutic effects when baseline performance is accounted for statistically, highlighting the clinical relevance of both taping methods as adjuncts to rehabilitation.

### 4.4. Limitations and Implications for Future Research

The primary limitation of this study was the reliance on clinical assessments rather than objective measurements, such as those provided by advanced devices. Future studies should incorporate objective tools to validate and expand upon these findings. A key limitation of this study is the absence of a no-taping control group (e.g., sham taping or standard therapy), which limits the ability to isolate the specific effects of KT and RT. Future studies should include such a comparator to better distinguish true treatment effects from nonspecific or placebo responses. Another limitation was the different distribution of gender between groups. Future studies should also evaluate the extent to which gender distribution affects the results using factor analysis.

By comparing the long-term effects of KT and RT, the present study provides valuable insights into taping interventions for cerebral palsy and offers a foundation for future research to optimize therapeutic strategies.

## 5. Conclusions

The findings of this study suggest that both KT and RT interventions significantly enhance gross motor function, static and dynamic balance, and functional capacity in children with HCP. These results highlight the efficacy of KT and RT as valuable therapeutic approaches for the rehabilitation of children with HCP. Incorporating these interventions into rehabilitation programs may provide complementary benefits, ultimately improving the overall functional outcomes in this population. These findings indicate that KT and RT are similarly effective adjuncts to physiotherapy in the rehabilitation of children with HCP, supporting the clinical applicability of both taping methods. The choice between KT and RT may therefore be guided by clinical preference, child tolerance, and therapeutic goals rather than superiority of effect.

## Figures and Tables

**Table 1:** Physical characteristics of the participants ^a^.

	KT Group (*n* = 26)	RT Group (*n* = 26)	*p* ^b^
**Age (y)** **Mean (SD)**	10.00 (±2.24)	9.5 (±2.94)	0.817
**Height (cm)** **Median (25–75%)**	129.53 (120.75–138.25)	131.03 (117.75–146.25)	0.095
**Weight (kg)** **Median (25–75%)**	31.73 (25.0–37.5)	29.96 (22.0–38.25)	0.327
**BMI (kg/m^2^)** **Median (25–75%)**	18.74 (15.35–19.90)	17.01 (15.30–18.83)	0.191
**Sex**	***n*** **(%)**	***n* (%)**	0.777
Female	10 (38.5)	11 (42.3)	
Male	16 (61.5)	15 (57.7)	
**Hemiplegic side**			0.710
Left	14 (53.8)	15 (57.7)	
Right	12 (46.2)	11 (42.3)	
**GMFCS**			0.405
Level I	15 (57.7)	12 (46.2)	
Level II	11 (42.3)	14 (53.8)	

^a^ Values are median (25th, 75th centile) for continuous variables and frequency for categorical variables. ^b^ Mann–Whitney *U* test for continuous variables and the Chi-Square test for categorical variables. KT, Kinesio Taping; RT, Rigid Taping; BMI, Body Mass Index; GMFCS, Gross Motor Function Classification System.

**Table 2:** Comparison of the treatment values within groups and the baseline scores of groups.

KT Group (*n* = 26)					RT Group (*n* = 26)				Comparison of Baseline Scores	
	Before Median (25–75%)	After Median (25–75%)	Eta Squared * (η^2^) *	*p*-Value ^a^	Before Median (25–75%)	After Median (25–75%)	Eta Squared * (η^2^) *	*p*-Value ^a^	Z	*p*-Value ^b^
**GROSS MOTOR FUNCTION**										
GMFM D (%)	92.30 (84.61–97.43)	94.87 (87.17–98.32)	0.45	**<0.001** **^a^**	92.30 (87.17–97.43)	92.30 (89.74–97.52)	0.57	**<0.001 ^a^**	−0.09	0.92
GMFM E (%)	89.57 (83.33–95.80)	90.27 (86.80–97.56)	0.63	**<0.001** **^a^**	90.27 (87.50–93.05)	91.66 (88.53–94.40)	0.75	**<0.001** **^a^**	−0.00	0.99
**BALANCE**										
Berg Balance Scale (p)	49.00 (48.00–52.25)	50.00 (48.00–53.00)	0.41	**<0.001** **^a^**	49.00 (47.75–52.00)	51.00 (49.00–53.00)	0.54	**<0.001** **^a^**	−0.426	0.67
TUG (s)	6.94 (6.35–8.35)	6.31 (5.93–7.99)	0.56	**<0.001** **^a^**	8.06 (7.62–8.69)	7.77 (7.24–8.39)	0.19	**<0.001** **^a^**	−2.178	0.22
**FUNCTIONAL CAPACITY**										
2 min walking (m)	131.83 (123.13–136.66)	136.88 (125.06–140.72)	0.70	**<0.001** **^a^**	124.89 (121.40–131.67)	128.32 (123.06–132.37)	0.28	**<0.001** **^a^**	−1.464	0.14

Values are median (25th, 75th centile). ^a^ *p*-value for intra-group change calculated using the Wilcoxon signed-rank test. ^b^ *p*-value for the inter-group difference in baseline scores calculated using Mann–Whitney *U* tests. Statistically significant at *p* < 0.001. KT, Kinesio Taping; RT, Rigid Taping; GMFM, Gross Motor Function Measurement; TUG, Time and Go Test. %, percentage; p, point; s, second; m, meter. They are marked in bold because there are significant differences.

**Table 3:** Comparison between the groups in terms of post-intervention change.

Between Baseline and 12 Weeks ^a^	KT Group Median Differences (25–75%)	RT Group Median Differences (25–75%)	Eta Squared (η^2^)	Z	*p*-Value ^b^
**GROSS MOTOR FUNCTION**					
GMFM D (%)	1.38 (0.00–2.57)	2.29 (0.00–2.57)	0.0004	−0.138	0.890
GMFM E (%)	1.39 (1.06–2.78)	1.39 (1.29–1.39)	0.0005	−0.156	0.876
**BALANCE**					
Berg Balance Scale (p)	0.50 (0.00–1.25)	2.00 (0.00–2.00)	0.003	−0.370	0.711
TUG (s)	−0.52 (−0.66–0.31)	−0.14 (−0.58–0.10)	0.14	−2.709	**<0.001**
**FUNCTIONAL CAPACITY**					
2 min walking (m)	3.69 (1.70–4.95)	1.63 (−0.5–3.65)	0.085	−2.096	**0.036**

^a^ Post-intervention change is calculated by subtracting the baseline value from the post-session value. ^b^ *p*-value for inter-group differences calculated using Mann–Whitney U tests. Statistically significant at *p* < 0.05. , KT, Kinesio Taping; RT, Rigid Taping; GMFM, Gross Motor Function Measurement; TUG, Time and Go Test. %, percentage; p, point; s, second; m, meter. They are marked in bold because there are significant differences.

## Data Availability

The original contributions presented in this study are included in the article. Further inquiries can be directed to the corresponding author.
